# Estimating Molar Root Volume from Panoramic Radiographs Using a Geometric Approach—An Experimental Method Comparison

**DOI:** 10.3390/medicina61071261

**Published:** 2025-07-11

**Authors:** Katharina Hartmann, Markus Tröltzsch, Sven Otto, Matthias Tröltzsch

**Affiliations:** 1 Department of Oral and Maxillofacial Surgery and Facial Plastic Surgery, University Hospital, LMU Munich, Lindwurmstraße 2a, 80337 Munich, Germany; katharina.hartmann53@gmail.com (K.H.); sven.otto@med.uni-muenchen.de (S.O.); 2 Center for Oral, Maxillofacial and Facial Reconstructive Surgery, Maximilianstraße 5, 91522 Ansbach, Germany; troeltzsch@gmx.net

**Keywords:** panoramic radiography, cone-beam computed tomography, dental roots, mathematical models, alveolar process

## Abstract

*Background and Objectives*: Evaluating jaw augmentation procedures usually necessitates pre- and postoperative tomographic imaging. Ethical considerations emphasize minimizing radiation exposure. Given that panoramic radiographs (PR, 2D) offer a lower radiation dose compared to cone-beam CT (CBCT, 3D), this study explores the feasibility of estimating tooth root volume from PR, potentially allowing safer clinical assessments with reduced radiation exposure. *Materials and Methods*: To develop a mathematical approximation method, the 2D tooth root surface in PR was defined as an elliptical model and a cuboid (3D). The true root volume (mm^3^) was gathered from CBCTs. The missing link for tooth root volume assessment in 2D radiographs is the depth of the root (vestibulo-oral dimension). It was hypothesized that the tooth root surface and its volume are related. A correlation factor “r” corresponding to the tooth roots’ depths was then calculated. Descriptive and inferential statistics were computed (*p* < 0.05). *Results*: The mathematical model was performed on 27 molars with an average volume of 472.83 mm^3^ (±130.25–CBCT). The factor “r” (obtained by dividing the true root volume from CBCT by the total root surface from PR) was computed as 8.04 (±1.90). Using “r” for the volume calculation in the cuboid model, an average volume of 472.37 (±152.92) for the 27 molars was computed. These volumes did not differ significantly. *Conclusions*: This study demonstrates that a mathematical model using elliptical projections from panoramic radiographs reliably estimates molar root volume, yielding comparable results to CBCT while reducing radiation exposure.

## 1. Introduction

Implantology relies on precise planning procedures, advanced surgical techniques, and innovative materials to ensure the long-term success of dental implants [1]. Computer-assisted design and manufacturing of implant placement splints have also considerably simplified surgical procedures [2]. However, a sufficient bone volume at the implant site is a prerequisite for successful implantology [3,4]. Insufficient bone volume not only jeopardizes primary stability and osseointegration but also prevents the implant from being placed in the optimal prosthetic position, which may complicate or preclude the restoration [5,6,7].

Various techniques to enhance bone volume prior to implant placement are used in clinical practice when significant resorption has taken place [8,9]. However, the main goal should be to prevent jawbone resorption and avoid complex, expensive, and time-consuming augmentation procedures before implant placement. Ridge preservation techniques may help maintain bone volume after tooth extraction [10,11,12,13]. They can be performed immediately after tooth extraction and are not very technically challenging [14]. Therefore, these procedures are frequently performed. A variety of different augmentation materials for ridge preservation are available (autologous, allogenic, xenogenic) [10,11,15].

Surprisingly, the literature on the complications and success rates of simple ridge preservation procedures is scarce. The degree of bone ingrowth into the augmentation materials, the time until bony consolidation at the site, and the volume stability of the procedure are largely unknown [16,17,18]. Ethical, methodological, and technical issues are responsible for the lack of this knowledge [13]. To further elucidate those matters, much more research will be necessary.

Tomographic radiological imaging is essential for research on alveolar bone augmentation techniques and volume assessment [12]. Segmentation procedures can be used to define regions of interest, which are the basis of volume calculation. To assess the efficacy of ridge preservation procedures (defined as complete fill of the alveolar socket with bone after tooth extraction), the preoperative defect volume and postoperative bone volume must be known. If a pre-augmentation volume assessment cannot be performed (e.g., due to a lack of radiological data), the post-augmentation volume is not meaningful.

Tomographic imaging (i.e., CBCT or CT) is not usually indicated before tooth extractions and ridge preservation. However, panoramic radiographs (PR) combine minimal radiation exposure with sufficient clinical information [19] and are therefore frequently obtained [20] before tooth extractions. The radiation dose applied by PR is approximately 10–30 µSv. CBCT delivers 3 to 10 times more radiation, depending on the protocol used. Even in a scientific study setting, obtaining preoperative tomographic images before simple dentoalveolar surgery is debatable due to ethical reasons. Patients must not be exposed to unnecessary radiation [21,22]. There is a need for alternative analytical methodologies capable of accurately quantifying the preoperative bone defect volume after tooth extraction (ideally the volume of the alveolus), even if tomographic images are not available [23]. Such methods would enable a more efficient and straightforward process for volume analysis in research related to tooth root volume and ridge preservation. In the clinical setting, the methods can be used to estimate the volume of the defect following extraction and the necessary amount of bone graft material needed during ridge preservation.

The present study aims to propose a novel method for assessing molar root volume using PR. Given the minimal extent of the periodontal ligament, there is some similarity between the alveolar socket volume and the root volume. To guide this investigation, the following research question was formulated: Can the volume of tooth roots be extrapolated from their radiological appearance in PR? It was hypothesized that a strong correlation exists between the root surface measured in 2D radiographs (PR) and the true root volume as determined in CBCT.

## 2. Materials and Methods

### 2.1. Study Design

To address the research question, a proof-of-concept method comparison study protocol was designed and implemented. Appropriate cases from a cohort of patients who underwent molar exodontia and consecutive ridge preservation procedures, and who had preoperative PR and CBCT, were selected. The recording technique of PRs involves a distortion factor that is minimal in molar sites [20]. Therefore, only molar tooth roots were included in the data analysis. Periodontal attachment loss or furcation involvement, as well as excessive periapical radiolucencies (>5 mm in diameter), were exclusion criteria. These criteria were selected to minimize the risk of excessive measurements in situations with unclear radiological borders and to reduce the risk of root volume overestimation. The radiographs and data used for this study were irreversibly anonymized prior to the scientific analysis. According to the regulations of the ethics committee of the LMU Hospital, analysis of such data is exempt from a formal ethical consultation (Ethics Committee LMU Munich 24-0960-KB).

### 2.2. Radiologic Analyses and Variables

**Figure 1:**
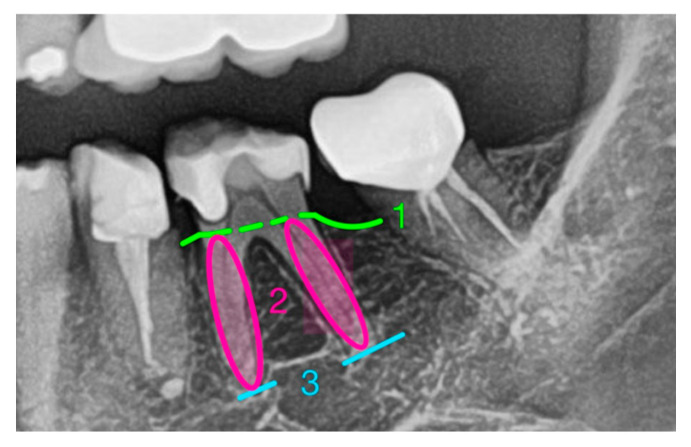
(1) crestal bone margin, (2) elliptical shape of the root surface, (3) root apex.

The morphology of tooth roots is unique, and various shapes have been described [24]. In physical experiments, the resemblance of the tooth root surface to an ellipse has been pointed out [25]. This model was adopted in this study, and the 2D surface of the tooth roots was defined as having an elliptical shape with a clear mathematical definition (surface area: A = π × a × b; Figure 1). This formula provides an approximation for the root surface area estimated from panoramic radiographs.

**Figure 2:**
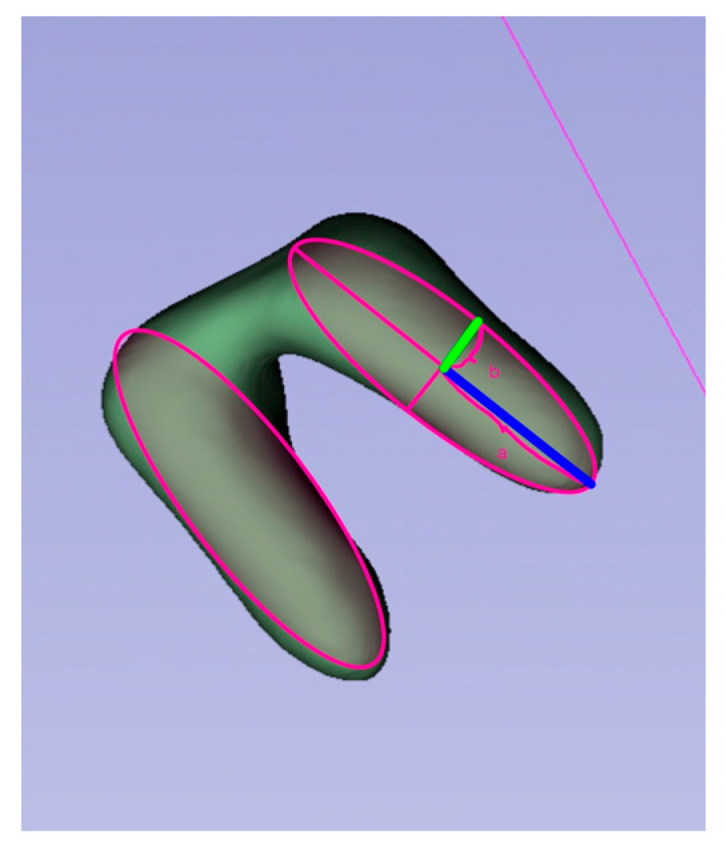
(a, blue line) half of the root length; (b, green line) half of the root width (root model exported from Slicer 3D^®^ (Slicer 5.3.0, Brigham and Women’s Hospital, Boston, MA, USA) for illustration purposes.

The variable a describes exactly half of the ellipse’s length, and the variable b describes half of its width (Figure 2).

**Figure 3:**
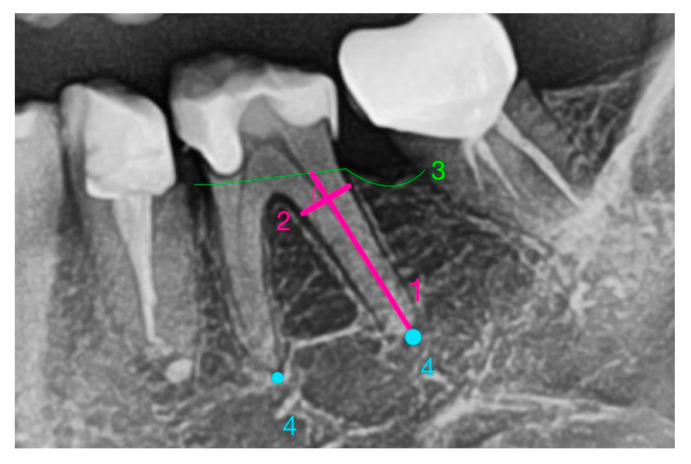
(1) root length, (2) root width at the root’s widest part, (3) crestal bone margin, (4) apex; 1 and 2 are perpendicular to one another.

In this proposed model, the variable a is defined as half the length of the root (total root length measured from the most apical point of the root tip to the crestal bone margin). The variable b is defined as half the width of the widest part of the root, measured perpendicular to the length axis, regardless of its coronal-apical position within the alveolus (Figure 3).

All measurements of the root length and width on the PRs were conducted using the measuring tape tool of the byzz^®^ Nxt (Orangedental, Version 10.2.142, 2024) radiological software. The measurements were done by two operators simultaneously.

**Figure 4:**
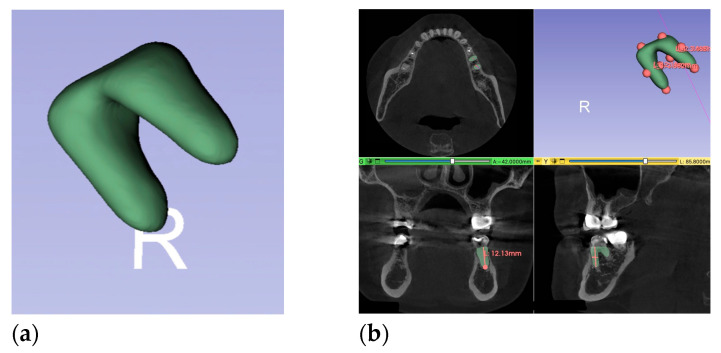
(**a**) tooth root from the CBCT with 3D-Slicer; (**b**) root in all axes in the CBCT with length, width, and depth measurements.

The true root dimensions and volume were gathered by (volumetric) analysis of the corresponding CBCT images. For the assessment of the root volume in CBCTs, the pre-validated open-source program 3D Slicer ^®^ (Brigham and Women’s Hospital, Boston, MA, USA) was used [26]. Two segments, “alveolus” and “alveolar ridge”, for each tooth were created. The “alveolus” was defined as the body of the tooth at the crestal bone level, and the “alveolar ridge” as the surrounding structure in the CBCT. The “grow from seed” function (seed size 2.3) was used to fill any gaps. This validated software function allows for the reduction of operator-dependent inaccuracies. After the segmentation process, a 3D model of the alveolar shape was generated, and the real length and width were measured using the above-mentioned definitions (Figure 4). Furthermore, the bucco-lingual diameter (root depth) of the 3D model was determined. The rough surface was refined by removing extra parts using the “island” tool, filling holes, and manually trimming errors with “scissors,” followed by applying “joint smoothing” (smoothing factor 46) until a smooth model was achieved. The volume of the 3D model was computed in 3D Slicer^®^.

All the gathered values (length, width from CBCT and PR for mesial and distal roots; root depth and volume from CBCT) were recorded. The elliptical surface areas computed from PR and CBCT measurements were also tabulated. The complete root surface was defined as the addition of the elliptical surface areas of the mesial and distal roots.

### 2.3. Simplified Geometric Approximation Method for the Tooth Root Volume

To create a volumetric model that can be developed from a known surface area (as to be obtained in the 2D PR), the dental root was approximated as a cuboid in this study, despite its inherent anatomical complexity. Although dental roots are naturally tapered and irregular, using simplified geometric forms is common in computational dental analysis when the goal is to standardize comparisons across cases [27,28]. A geometrical approximation of the tooth root volume has not been previously suggested. However, similar theoretical algorithms for root canal volume determination have been proposed before [28,29,30]. Due to the inherent limitations of panoramic radiographs in capturing continuous taper, tooth roots were approximated as cuboids based on measurable elliptical surface areas.

A cuboid volume can be calculated from its base area and depth (Volume V = base area A × depth d). The base area of the cuboid root volume model in this study was defined as the elliptical surface area.

In a first step, data from the CBCTs (length, width, and depth) were used to calculate the approximated cuboid model volume. This was then compared to the true volume of the alveolar model as gathered from the segmentation process in 3D Slicer^®^. The average values of the cuboid volume and root volumes were calculated.

In a second step, a constant that substitutes the value d (depth) for calculations in PRs had to be established. The formula for the cuboid volume, V = A × d, can be rearranged to solve for d when the values of the base area A and the total volume V are known. The resulting equation is d = V/A. The true volume was gathered from the 3D model, and the approximated base area (addition of the elliptical surfaces of the mesial and distal roots obtained from PR) was divided from it. This resulted in a hypothetical constant that may depict the roots’ average depth in approximation. This hypothetical constant was labeled the projection factor r.

In a third step, the validity of this concept had to be proven. Using the formula V = A × r, the approximated volumes for the molar roots were computed for each individual case. These volumes were then compared to the true volume (3D model) and the approximated cuboid (values from CBCT measurements). Each volume was then back-checked using r and the CBCT-derived true diameter d.

The predictor variable was the true volume (3D model) as determined through the segmentation process in 3D Slicer^®^. The outcome variable was the extrapolated volume calculated by multiplying the elliptical root surface in PR by the projection factor r. The projection factor r—calculated as the ratio of CBCT-derived volume to PR-derived elliptical surface area—was applied as a constant across cases after preliminary analyses indicated minimal variability within the sample.

### 2.4. Statistical Analysis

The gathered data were tabulated and statistically analyzed using SPSS for Mac^®^ (Version 30.0, IBM, Armonk, NY, USA). Descriptive and inferential statistics were computed. Normality of the data was confirmed via Kolmogorov–Smirnov tests (*p* > 0.05). Differences between groups were assessed using pairwise t-tests with a significance level of *p* < 0.05. Effect sizes were determined using G-Power for Mac (Version 3.1.9.6, Heinrich-Heine University of Düsseldorf, Germany). Inherent to our proof-of-concept design, a post hoc power analysis was conducted, and Bayesian analyses were performed using JASP for Mac (Release 0.19.3, University of Amsterdam, The Netherlands) to complement the frequentist methods by providing robust parameter estimates and credible intervals in case of limited effect sizes.

## 3. Results

The study sample was composed of 27 individual patient cases. Both panoramic radiographs and CBCT datasets were evaluated. The measured values of root lengths/widths/elliptical surface areas and total volumes as gathered from panoramic radiographs and CBCTs are displayed in Table 1 (all linear measurements are in mm, all surface measurements are in mm^2^, and all volumes are in mm^3^). The mathematical algorithm described above was applied to 27 individual patient cases. Both panoramic radiographs and CBCT datasets were evaluated. The measured values of root lengths/widths/elliptical surface areas and total volumes as gathered from panoramic radiographs and CBCTs are displayed in Table 1 (all linear measurements are in mm, all surface measurements are in mm^2^, and all volumes are in mm^3^). 

The normal distribution of the gathered values for length, width, surface area, and volumes obtained from PR and CBCT was confirmed (Kolmogorov–Smirnov Test for all groups, *p* > 0.05). The variables were analyzed for differences using a pairwise *t*-test. Length measurements between PR and CBCT showed significant differences (*p* < 0.03 mesial and *p* < 0.01 distal), whereas the width measurements did not (*p* > 0.9 mesial and *p* < 0.6 distal). The average root depth as measured on CBCT was 7.9 mm for the mesial root and 7.8 mm (±2.3 mm) for the distal root. As shown in Table 1, the average true root volume was 472.83 mm^3^ (±130.25). All values were strongly and significantly correlated with Pearson correlation coefficients of 0.7 and above (all *p* values < 0.01).

The total elliptical surface areas obtained from PR and CBCT showed significant differences (*p* < 0.014). As described above, the projection factor r was calculated by dividing the true root volume by the total elliptical surface area (obtained from PR and defined as the base area) for all cases, averaging 8.04 (dimensionless). Although the total elliptical surface area (the sum of the elliptical surfaces of both roots) from PR and CBCT measurements exhibited statistically significant differences (*p* > 0.02), the true root volume (obtained from CBCT) and the extrapolated r-dependent volume from PR did not show significant differences (*p* > 0.41).

The CBCT root measurements (length/width/depth) were used to calculate the volume of the cuboid model per tooth, which was 536.88 mm^3^ (±171.64), significantly differing from the true root volume (*p* < 0.013) and the extrapolated root volume based on the PR-based cuboid volume model (*p* < 0.02).

Table 2 summarizes the average values of the CBCT-based cuboid model, the average surface area for the mesial and distal roots in the elliptical model as obtained from CBCT, and the consecutive calculated volumes.

**Figure 5:**
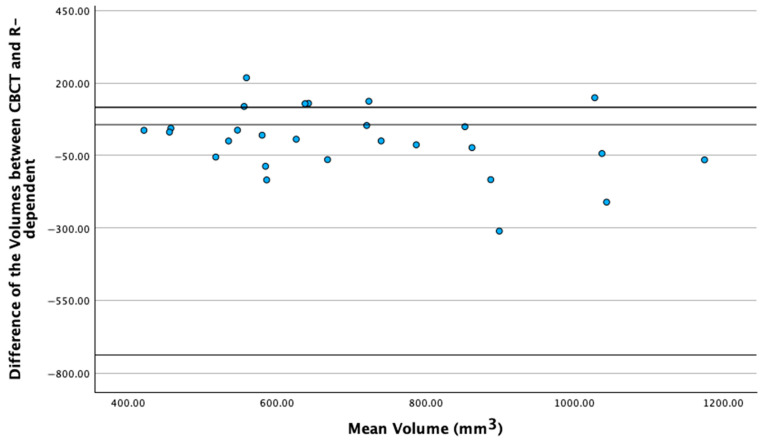
Bland–Altman plot comparing PR-based cuboid model volumes with CBCT-derived true root volumes.

To evaluate the validity of the proposed tooth root volume determination model, a post-hoc power analysis was performed. The mean difference between the true CBCT-based measurements and the projection factor PR-based measurements was 0.0008 (post-hoc, differences between two dependent means of matched pairs, mean volume CBCT, and mean PR volume with r displayed in Table 1, sample size 27, correlation calculated with the Pearson correlation coefficient 0.7). The analysis yielded a statistical power of 5% due to the very low effect size. In order to achieve a statistical power of 80% a sample size of 11,500,640 would be necessary. Bayesian methods were employed to further clarify the strength of the proposed model. In the Bayesian linear model assessing whether the 2D radiographic root surface predicts true root volume, the intercept is estimated at 139.19 (SE = 66.16) with a 95% credible interval of [5.25, 269.65]. The convergence diagnostics—specifically, an R-hat value of 1.00 and effective sample sizes exceeding 6000 for the central posterior estimates and around 3900 for the tails—demonstrate that the Markov Chain Monte Carlo (MCMC) sampling converged reliably, ensuring robust parameter estimation. This Bayesian analysis shows that the PR-based measurements predict true CBCT volumes with high confidence because the probability intervals around the key coefficients are narrow. Thus, it can be inferred that the estimated relationship is both strong and reliable. In the Bland–Altman plot (Figure 5), although the PR-based volumes show a mean bias of −311.29 mm^3^ with 95% limits of agreement from −738.65 to 116.07 mm^3^ indicating a systematic underestimation compared to CBCT, the strong Pearson correlations (r > 0.7) along with favorable Bayesian analysis confirm that the model reliably captures volumetric trends, supporting its feasibility as a proof-of-concept despite the observed bias.

## 4. Discussion

The aim of the present study was to examine whether two-dimensional radiographs, such as panoramic radiographs, can be used to assess the root volume of molar teeth and whether tooth root volume could be inferred from their appearance in panoramic radiographs. The concept was developed because CBCT obtainment is not possible or not indicated in most cases of dentoalveolar surgery. The concept development followed two main ideas: First, the method could have clinical value as it allows for estimating the necessary amount of augmentation material for ridge preservation, thereby reducing the disposal of excess material, which has environmental and economic benefits. Second, it might also hold scientific significance. In retrospective studies, it may be intriguing to understand the pre-extraction alveolar volume to gauge the success of alveolar ridge preservation. The hypothesis was that the shape of the roots and their volume are related.

The findings indicate that the root volume (of molar teeth) can be approximated based on its elliptical surface area, which can be obtained from panoramic radiographs when multiplied by an artificially created projection factor “r.” Furthermore, there is a strong correlation between the root surface area (assuming elliptical shapes as described in Section 2) and the true root volume as gathered from CBCT models.

The study showed that PR measurements correlate well with CBCT measurements concerning root length and width. However, the length measurements on PR differed significantly from the CBCT measurements, while the width measurements did not. These measurements were used to calculate the approximated two-dimensional surface areas, which were assumed to be elliptical. The surface areas obtained from PR measurements and CBCT measurements differed significantly. Once again, the results demonstrated high correlation coefficients (Pearson correlation coefficient > 0.7).

Despite the differences in measurements, the extrapolated root volume calculated from PR measurements using the artificially created projection factor did not significantly differ from the true root volume (*p* > 0.412). This suggests the applicability of the proposed model. Furthermore, it may show that slight inaccuracies of length/width measurements in PR might eventually have little to low influence on the correct volume estimate. Although the proposed cuboid-based algorithm and molar-specific calibration streamline volumetric estimates for clinical and research use, the geometric simplification and focus on molars may limit accuracy for non-molar or highly curved roots, indicating a need for extension and revalidation across diverse dental morphologies. The sole cuboid model’s 13% overestimation of root volume (536.88 mm^3^ vs. 472.83 mm^3^) without the use of the projection factor r suggests that using unadjusted PR-based predictions in surgical planning may lead to excess bone graft harvesting or the choice of excess bone graft, resulting in possibly higher perioperative morbidity and increased costs.

Literature searches revealed only one other study that attempted to estimate the volume of tooth roots from PRs [23]. Extensive database and manual searches revealed no other relevant investigations into PR-based root volumetry, highlighting the pioneering nature of this methodology and the need for future exploration.

In this study, the authors measured the tooth roots and crowns in PRs and CBCTs and created a tooth model. The average difference between the tooth model from the panoramic radiograph and the segmented tooth model from the CBCTs was 0.7 mm^3^ for the root area of molars. This suggests that the PR can provide enough information to estimate the volume of tooth roots. Although direct PR-to-root volumetry is novel, analogous 2D-to-3D approaches, such as cephalometric prediction of alveolar bone volume and radiograph-guided sinus segmentation, demonstrate the feasibility of reconstructing three-dimensional structures from planar images and provide a valuable framework for further refining our cuboid-based molar volume model [31].

The following limitations of this study need to be acknowledged. This study serves as a proof of principle and is the only one of its kind in the literature. The sample size is limited and insufficient to adequately demonstrate its validity with sufficient power. R was derived and re-checked using the same sample, which might further reduce its generalizability and validity. However, the study is innovative and is the first to suggest the applicability of two-dimensional radiographs for volumetric analyses. The sample size required to achieve a power of 80% cannot be reached due to the minimal effect size. The Bayesian model was developed to evaluate the quality of the proposed algorithm and confirmed its validity. R was checked for each single case, and the volume calculations remained constant. Although the pairwise t-test revealed no significant differences between PR-based and CBCT-based volumes (*p* > 0.412), the low statistical power (5%) of our small, ethically constrained sample necessitates cautious interpretation; nevertheless, robust Bayesian analysis and high correlation coefficients (r ≥ 0.7) substantiate the reliability of our volumetric estimation model.

The suggested volumetric tooth root model, designed as a cuboid, contains inaccuracies and can only provide a rough approximation. Nevertheless, the results show that the model correlates well with the true root volume, and the measurements do not significantly differ from the actual volumes. The model was tested only for molar roots. Due to the overprojection of the palatal roots, the root volume assessment of first maxillary premolars and maxillary molars will be inaccurate with this model. Finally, it must be acknowledged that PRs only contain (distorted) 2D data. Therefore, the results obtained cannot be considered equal to those from CBCT-based 3D data.

## 5. Conclusions

Within the limitations of this study, it can be concluded that PRs (two-dimensional radiographs) have some value in the assessment of molar root volumes based on a cuboid volumetric model and an artificial projection factor.

## Figures and Tables

**Table 1:** (**A**,**B**). Values of all length, width, surface, and volume measurements; the mean values and standard deviations are displayed in the last line in bold letters. (**A**): linear measurements (mm). (**B**): surface measurements (mm^2^) and volumetric measurements (mm^3^).

(**A**)							
**Mesial Root Length PR**	**Mesial Root Length in CBCT**	**Mesial Root Width PR**	**Mesial Root Width CBCT**	**Distal Root Length PR**	**Distal Root Length CBCT**	**Distal Root Width PR**	**Distal Root Width CBCT**
11.06	12.13	2.53	3.88	11.97	10.61	2.52	3.49
12.06	13.74	2.88	2.57	10.40	10.85	3.59	4.07
16.66	16.07	3.96	4.02	15.90	15.17	4.20	4.52
12.44	14.16	3.91	3.02	12.90	14.83	3.29	3.70
14.50	13.82	4.58	4.63	13.44	13.12	3.72	4.64
11.69	12.81	3.14	3.39	11.58	12.76	3.86	3.53
13.00	12.61	4.30	4.54	13.78	13.81	5.11	5.36
12.72	12.84	3.51	3.91	12.16	11.57	3.51	5.33
10.43	11.82	5.07	4.26	7.11	11.12	3.30	4.42
8.52	9.03	3.49	4.54	7.38	8.76	3.95	3.28
11.34	13.46	4.27	5.89	13.84	13.55	4.07	6.08
10.06	11.10	3.03	4.06	10.80	10.07	3.58	4.40
7.62	10.37	2.60	3.23	9.77	10.11	2.88	2.65
8.72	10.90	3.01	4.09	6.06	6.98	2.99	3.98
11.91	8.75	3.88	3.03	9.11	8.61	2.82	2.24
9.37	8.87	3.06	3.33	8.50	10.01	2.33	3.77
11.98	11.57	4.50	2.69	13.73	11.79	4.52	3.52
9.64	7.95	3.09	3.84	8.45	9.72	2.94	4.59
9.10	14.16	3.34	4.17	8.59	12.33	3.86	3.93
10.06	10.71	4.65	3.56	8.36	9.00	3.62	3.55
10.43	11.83	4.47	3.05	9.02	9.16	2.43	3.59
12.31	12.14	3.07	3.26	9.56	10.76	2.61	4.07
8.97	9.93	2.99	2.71	8.39	10.54	3.96	3.62
9.75	9.66	3.48	3.30	8.95	12.63	3.26	3.07
9.86	10.90	7.77	4.00	9.38	11.17	3.02	3.80
8.37	10.17	2.99	3.32	7.47	8.34	3.28	3.66
12.72	12.71	3.43	4.14	12.23	12.19	4.29	3.67
**10.94 ± 2.04**	**11.64 ± 1.95**	**3.74 ± 1.05**	**3.71 ± 0.93**	**10.33 ± 2.51**	**11.09 ± 2.02**	**3.46 ± 0.67**	**3.94 ± 0.81**
(**B**)							
**Elliptical Surface Both Roots PR**	**Elliptical Surface Both Roots CBCT**		**Projection Factor r = V_total_/A_surface_ _PR_**		**Projection Factor r-Dependent Volume PR**	**Volume with Cuboid Model CBCT Measurements**	**True Root Volume CBCT**
45.67	66.05		8.53		352.56	362.22	389.73
56.60	62.48		10.14		436.97	412.76	573.76
104.26	104.51		7.09		804.92	711.01	739.55
71.54	76.69		8.41		552.25	559.66	601.36
91.43	98.03		7.24		705.81	712.38	662.27
63.94	69.54		7.72		493.58	452.20	493.58
99.21	103.05		5.59		765.89	781.99	554.57
68.59	87.83		7.52		529.50	569.44	516.09
59.96	78.17		8.61		462.89	704.22	516.16
46.25	54.71		7.72		357.04	581.22	356.85
82.27	126.97		9.53		635.13	1082.90	784.24
54.31	70.21		6.11		419.25	429.02	331.67
37.66	47.33		8.88		290.73	361.77	334.43
34.85	56.82		8.77		269.01	456.39	305.62
56.47	35.97		5.34		435.96	264.77	301.32
38.07	52.80		8.52		293.93	492.42	324.38
91.08	56.96		4.30		703.16	395.71	391.86
42.91	59.00		10.49		331.24	463.26	450.22
49.91	84.36		10.32		385.33	627.33	515.02
60.51	55.06		6.65		467.13	533.58	402.53
53.83	54.23		7.83		415.58	389.20	421.40
49.28	65.47		8.12		380.43	437.77	400.14
47.16	51.12		6.54		364.07	625.69	308.38
49.56	55.49		10.31		382.64	566.75	511.06
82.42	67.58		6.10		636.28	599.96	502.55
38.90	50.49		13.33		300.30	306.49	518.34
75.47	76.46		7.41		582.66	615.93	559.49
**61.18 ± 19.80**	**69.16 ± 20.71**		**8.04 ± 1.9**		**472.37 ± 152.92**	**536.88 ± 171.64**	**472.83 ± 130.25**

**Table 2:** Average values of true root volumes, CBCT-based elliptical total surface areas, r, and r-dependent PR-based root volume.

Average Surface of Both Ellipses (mm^2^)	Factor “r”	Average Volume Calculated with “r” (mm^3^)	Average Volume Booth Tooth Roots CBCT (mm^3^)	Pairwise *t*-Test
61.18 ± 19.80	8.04 ± 1.90	472.37 ± 152.92	472.83 ± 130.25	*p* > 0.412

## Data Availability

The datasets used and analyzed during the current study are available from the corresponding author on reasonable request.

## Abbreviations

CBCT	cone-beam computer tomography
PR	panoramic radiographs
